# Alleviating Nitrogen and Phosphorus Limitation Does Not Amplify Potassium‐Induced Increase in Terrestrial Biomass

**DOI:** 10.1111/gcb.70193

**Published:** 2025-04-23

**Authors:** Guopeng Liang, Pengyan Sun, Bonnie G. Waring, Zheng Fu, Peter B. Reich

**Affiliations:** ^1^ Department of Forest Resources University of Minnesota St. Paul Minnesota USA; ^2^ Department of Ecology and Evolutionary Biology Yale University New Haven Connecticut USA; ^3^ Institute for Global Change Biology and School for Environment and Sustainability University of Michigan Ann Arbor Michigan USA; ^4^ School of Statistics University of Minnesota Minneapolis Minnesota USA; ^5^ Grantham Institute on Climate Change and the Environment and the Georgina Mace Centre for the Living Planet Imperial College London London UK; ^6^ Key Laboratory of Ecosystem Network Observation and Modeling, Institute of Geographic Sciences and Natural Resources Research Chinese Academy of Sciences Beijing China; ^7^ Hawkesbury Institute for the Environment Western Sydney University Penrith New South Wales Australia

**Keywords:** nutrient limitation, plant productivity, potassium, root biomass, soil organic carbon, terrestrial ecosystems

## Abstract

Potassium (K) is the second most abundant nutrient element in plants after nitrogen (N), and has been shown to limit aboveground production in some contexts. However, the role of N and phosphorus (P) availability in mediating K limitation in terrestrial production remains poorly understood; and it is unknown whether K also limits belowground carbon (C) stocks, which contain at least three times more C than those aboveground stocks. By synthesizing 779 global paired observations (528, 125, and 126 for aboveground productivity, root biomass, and soil organic C [SOC], respectively), we found that K addition significantly increased aboveground production and SOC by 8% and 5%, respectively, but did not significantly affect root biomass (+9%). Moreover, enhanced N and/or P availability (through N and P addition) did not further amplify the positive effect of K on aboveground productivity. In other words, K had a positive effect on aboveground productivity only when N and/or P were limiting, indicating that K could somehow substitute for N or P when they were limiting. Climate variables mostly explained the variations in K effects; specifically, stronger positive responses of aboveground productivity and SOC to K were found in regions with high mean annual temperature and wetness. Our results suggest that K addition enhances C sequestration by increasing both aboveground productivity and SOC, contributing to climate mitigation, but the positive effects of K on terrestrial C stocks are not further amplified when N and P limitations are alleviated.

## Graphical abstract

By collecting and analyzing the data from 779 global paired observations, we found that potassium addition can increase aboveground production and soil organic carbon content by 8% and 5%, respectively, and thus contribute to terrestrial carbon sequestration. However, applying nitrogen and phosphorus does not further amplify the positive effects of potassium addition on aboveground production and soil organic carbon. Our study suggests that potassium addition plays a great role in stimulating terrestrial carbon stock, but this benefit is not enhanced by alleviating nitrogen and phosphorus limitation.

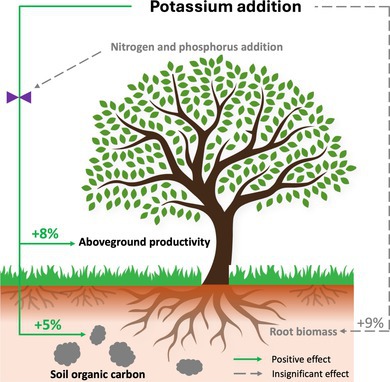

## 1 Introduction

Terrestrial ecosystems are crucial for global climate mitigation, biodiversity, and food production (Griscom et al. 2017; Manning et al. 2018); however, their provision of these ecosystem services may be constrained by nutrient limitations. For example, multiple studies have found that both nitrogen (N) and phosphorus (P) can stimulate aboveground production (Lebauer and Treseder 2008; Du et al. 2020; Hou et al. 2020), indicating widespread N and P limitation of terrestrial ecosystems. Additionally, this likely also means that N and P availability constrain the capacity of ecosystems to remove additional CO_2_ from the atmosphere (Reich and Hobbie 2013; Jiang et al. 2020; Terrer et al. 2021). Because of this, interactions among N, P, and carbon (C) cycles have been incorporated into some Earth System Models (ESMs) to more accurately predict future C‐climate feedbacks (Wang et al. 2010, 2020). Although potassium (K) is a key plant macronutrient required at the second highest concentration after N and the most abundant cation in plant cells (Dreyer and Uozumi 2011), its effects on plant community production and terrestrial C stocks have been largely overlooked (Sardans and Peñuelas 2015; Schlesinger 2021).

A previous meta‐analysis study on global K limitation in terrestrial ecosystems mainly focused on aboveground productivity under control and K addition (Chen et al. 2023). Given that 18% and 43% of the natural terrestrial land area are significantly limited by N and P, respectively (Du et al. 2020), the K addition experiments in the earlier study likely encompassed ecosystems with varying degrees of N and P limitations. This variability could lead to either an underestimation or overestimation of K effects on aboveground productivity at a global scale if the data points for the previous meta‐analysis (Chen et al. 2023) were not sampled evenly from the distribution of natural ecosystems. Moreover, uncertainty related to the relative co‐limitations by N and/or P inhibits our understanding of whether alleviating N and/or P limitations—through fertilization or deposition, as commonly observed in nutrient management practices and real‐world conditions—would further enhance terrestrial ecosystem responses to K addition. By collecting and analyzing datasets from fully factorial nutrient addition experiments, we herein directly test whether K effects on aboveground productivity could be further amplified once the primary limitations (e.g., N and P) are alleviated. This can improve our understanding of the interactive effects of macronutrients on terrestrial ecosystems, which have been overlooked by ecologists, and provide useful and practical information to help policymakers and landowners apply the appropriate nutrient management for maximum terrestrial C sequestration and climate mitigation. Given the dominance of N and P as limits to plant productivity and the frequent co‐limitation of N and P in terrestrial ecosystems as found by previous studies (Harpole et al. 2011; Du et al. 2020), we expect to see greater K impacts when N and/or P or both are non‐limiting (Hypothesis 1).

Hypothesis 1 *K effects on aboveground productivity can be amplified when alleviating N and P limitation*.

By influencing leaf anatomy, mesophyll diffusion of CO_2_, chloroplast ultrastructure, rubisco quantity and activity, and photoassimilate translocation, K can significantly alter plant photosynthesis (Tränkner et al. 2018). Additionally, K can affect stomatal control and water conductance and is an important osmolyte, making it an essential element for drought avoidance (Wang et al. 2013; Sardans and Peñuelas 2015). Overall, K plays many fundamental physiological and metabolic roles in plants (Sardans and Peñuelas 2021); therefore, not surprisingly, some in situ fertilization experiments found K limitation of aboveground production in grassland and forest ecosystems (Wright et al. 2011; Baribault et al. 2012). In contrast, at some other sites, aboveground production was not K‐limited, perhaps because it was relatively available compared to other more limiting nutrients (Sardans and Peñuelas 2015). These opposing results beg an important question: does K limitation of aboveground production vary predictably with climatic and/or soil variables, similar to what has been found for both N (Liang et al. 2021) and P (Hou et al. 2020)? For example, different responses of plants to K were observed in two grasslands with a large difference in mean annual precipitation (MAP). In a mesic grassland (MAP: 1522 mm) in the UK (Crowther et al. 2019), K addition increased aboveground net primary productivity (ANPP) by approximately 55%, but did not significantly affect ANPP in a semiarid grassland (MAP: 643 mm) in Australia (Crowther et al. 2019). The fact that K can be leached from both leaves and soils more easily than N and P (Sardans and Peñuelas 2015) may lead to more serious K limitation in wet regions (e.g., high MAP or wetness) (Hypothesis 2).

Hypothesis 2 *K effects on plant production depend on moisture regime*.

When compared to aboveground production, the response of belowground C stocks (e.g., root biomass and soil organic C [SOC]) to K is more uncertain due to the limited relevant empirical evidence. On the one hand, when aboveground production is stimulated by K addition, this should result in greater resource requirements. As a result, roots may grow faster to uptake more resources (e.g., nutrients and water) from soils to support aboveground growth. On the other hand, K is involved in many mechanisms related to water economy and osmotic homeostasis (e.g., maintaining cellular turgor and osmotic pressure, controlling water conductance and transpiration, improving root hydraulic conductivity, and regulating sap flow, membrane potentials, and stomatal opening) (Wang et al. 2013; Sardans and Peñuelas 2015, 2021). As a result, K should increase water use efficiency (water lost per C gained photosynthetically), even without water stress (Grzebisz et al. 2013; Sardans and Peñuelas 2021), which may in turn decrease root growth. Overall, we hypothesize that the two aforementioned opposing mechanisms could counteract each other, leading to an insignificant effect of K on root biomass in terrestrial ecosystems (Hypothesis 3).

Hypothesis 3 *K effect is greater for aboveground than belowground biomass*.

It should be noted that, given multiple potential mechanisms, our expected results are highly uncertain.

Unlike N and P, K is not a constituent of biomolecules (e.g., proteins, carbohydrates, lipids, and nucleic acids) and has high solubility (Sardans and Peñuelas 2015, 2021). As a result, it can be easily and rapidly leached from soil organic matter (Sardans and Peñuelas 2021), and K is not directly chemically involved in SOC stabilization. Therefore, the impact of K on SOC should mainly stem from its effects on plants (Bar‐Yosef and Ben Asher 2013) and soil microbes (Sardans and Peñuelas 2021). To the best of our knowledge, however, no relevant studies have been done to determine the effect of K on SOC at the global scale. Generally speaking, K may affect SOC in three major ways. First, in K‐limited ecosystems, K could increase aboveground production and thus litterfall, which in turn should increase SOC by enhancing C input into soils. Second, the potential change in root biomass caused by K addition could further affect SOC inputs via root exudates and root necromass. Third, these effects may be further mediated by soil microbes. For example, the changes in both litterfall and root biomass caused by K addition could significantly affect soil microbial communities and activity (e.g., soil priming effect) (Kuzyakov 2002, 2010). The effects of K addition on soil respiration can be positive (Paul et al. 2021) or insignificant (Powers and Salute 2011; Mori et al. 2019). Overall, it is difficult to make a priori prediction of SOC response to K, especially when considering the roles of soil microbes. Since litterfall is the main C source for some (largely forested) soils (Vitousek 1984) and can significantly increase SOC (Leff et al. 2012), we expect that there should be a positive relationship between the response ratios of litterfall (represented by plant production here) and SOC (Hypothesis 4).

Hypothesis 4 *Soil C response to K is a function of K effects on production*.

## 2 Materials and Methods

### 2.1 Data Collection

The Web of Science was used to search for papers. For K effects on plant productivity, we used keywords “potassium addition OR potassium fertilizer AND aboveground biomass OR belowground biomass OR root biomass OR primary productivity”; and for K effects on SOC, we used keywords “potassium addition OR potassium fertilizer AND soil carbon.” The dataset about K effects on aboveground productivity (100 studies), root biomass (100 studies), and SOC (100 studies) in grasslands from the Nutrient Network project in a previous paper was also used in this study (Crowther et al. 2019).

The following criteria were used to select the appropriate studies: (1) to remove the potential effects of N and P, the difference between K treatment and control should be only K addition (e.g., K vs. control; NK vs. N; PK vs. P; and NPK vs. NP); (2) studies should be conducted at field sites in natural ecosystems; (3) if SOC and other soil parameters were reported at multiple soil depths, we only included the data in surface soils (e.g., 0–10 or 20 cm); and (4) for studies reporting results at multiple time points, only the result at the last time point was included. By following the aforementioned criteria, 779 paired observations (aboveground production: 528 paired observations from 123 field sites; root biomass: 125 paired observations from 38 field sites; and SOC: 126 paired observations from 31 field sites) were collected at the global scale (Figures S1 and S2 and Table S1). For each study, means and standard deviations of aboveground production, root biomass, SOC, and the corresponding replication were collected, respectively. Although our collected aboveground production included two types (productivity and biomass), we found its response to K did not vary with the type (Figure S3). In addition, we extracted site location, climate variables including mean annual temperature (MAT) and precipitation (MAP), K fertilizer type and application rate, N and P application rates, experimental duration, ecosystem type in natural ecosystems (forests, grasslands, wetlands, and tundra), and initial soil variables (SOC, pH, soil bulk density [BD], clay content [Clay], and total and available N, P, and K) (Table 1). For studies that did not report MAT and MAP, we extracted them from the database at http://www.worldclim.org/, using latitude and longitude with the “geodata” package. If initial soil variables including SOC, total N (TN), pH, BD, and Clay were not provided in the papers, their values at 0–5 cm soil depth were extracted from the database named “SoilGrids” (https://www.isric.org/) using the “geodata” package, with the help of the latitude and longitude. Given the wide range in MAT, MAP is probably a poor indicator of water availability. An aridity index (AI = MAP − potential evapotranspiration) can be a better indicator of likely potential leaching than MAP per se. Therefore, we also extracted AI from the database at https://figshare.com/articles/dataset/Global_Aridity_Index_and_Potential_Evapotranspiration_ET0_Climate_Database_v2/7504448/4 using the latitude and longitude of the studies with the “raster” package.

**TABLE 1:** Summary of site characteristics and the correlations between predictor variables and potassium effects on plant production and soil carbon.

Parameter	Unit	Aboveground productivity (*N* = 528)				Root biomass (*N* = 125)				Soil organic carbon (*N* = 126)			
		Range	Mean	*N*	Cor.	Range	Mean	*N*	Cor.	Range	Mean	*N*	Cor.
MAT	^o^C	−6.0 to 28.0	9.26	495	ns	−1.4 to 27.0	12.7	125	ns	3.8–18.4	10.9	123	ns
MAP	mm year^−1^	226–6828	884	495	+ (*)	246–6828	1088	125	ns	246–1877	853	123	ns
AI		0.00–4.80	0.86	495	+ (**)	0.15–4.80	0.81	125	ns	0.15–2.32	0.74	123	ns
ISOC	g kg^−1^	2.9–478.0	91.4	481	ns	5–276	55.9	122	ns	11.6–260.2	68.9	115	ns
TN	g kg^−1^	0.1–11.7	4.8	477	+ (*)	0.8–9.9	4.1	118	ns	0.8–11.7	4.5	115	ns
TP	g kg^−1^	0.05–38.10	6.2	37	ns	NA	NA	0	NA	NA	NA	0	NA
TK	g kg^−1^	0.07–24.68	5.4	33	ns	NA	NA	0	NA	NA	NA	0	NA
AN	mg kg^−1^	8.0–373.3	130.9	12	ns	8.0	8.0	4	NA	NA	NA	0	NA
AP	mg kg^−1^	1.8–152.0	25.2	111	ns	1.8	1.8	4	NA	5.6–152.0	22.2	13	ns
AK	mg kg^−1^	2.4–456.0	128.6	48	ns	103	103	4	NA	88.4–323.0	174.3	13	ns
pH		4.0–8.0	5.8	488	ns	2.9–7.8	6.1	122	ns	4.6–7.8	6.1	115	ns
Clay	%	1.0–40.0	19.6	179	− (**)	1.0–40.0	22.9	102	ns	8–40	23.4	104	ns
BD	g cm^−3^	0.6–1.6	1.13	433	− (*)	0.8–1.5	1.22	118	ns	0.6–1.5	1.20	115	ns
N rate	kg ha^−1^ year^−1^	24–448	163.3	220	ns	100–125	101.7	55	ns	24–300	99.2	56	ns
P rate	kg ha^−1^ year^−1^	3–350	119.5	230	+ (**)	34–100	93.6	54	ns	3–100	88.4	59	ns
K rate	kg ha^−1^ year^−1^	5–520	140.3	513	ns	20–225	99.6	121	ns	7–225	104.7	123	ns
Duration	year	1–120	5.6	512	ns	1–20	4.1	121	ns	2–120	11.8	126	ns

*Note:* The response variable is the response ratio of carbon stock (RR).

Abbreviations: −: negative correlation; +: positive correlation; AI: Aridity Index; AK: initial soil available potassium content; AN: initial soil available nitrogen content; AP: initial soil available phosphorus content; BD: initial soil bulk density; Clay: initial soil clay content; Cor.: the correlations between predictor variables and K effects on terrestrial carbon stock based on the results from simple linear regressions; Duration: experimental duration; ISOC: initial soil organic carbon; K rate: potassium application rate; MAP: mean annual precipitation; MAT: mean annual temperature; N rate: nitrogen application rate; *N*: study number; NA: not applicable; ns: not significant; P rate: phosphorus application rate; pH: initial soil pH; TK: initial soil total potassium content; TN: initial soil total nitrogen content; TP: initial soil total phosphorus content.

* *p* < 0.05.

** *p* < 0.01.

To determine whether K can increase SOC by increasing aboveground production and root biomass and thus litterfall and root exudates, respectively, SOC under both control and K treatments was also recorded if applicable when collecting aboveground production (*N* = 116) and root biomass (*N* = 103). Engauge Digitizer software (Free Software Foundation, Boston, MA, USA) was used to extract the data presented in figure form.

### 2.2 Data Analysis

We calculated the natural log‐transformed response ratio (RR) to quantify K effects on plant production and SOC by using the following equation:

(1) $$\mathrm{RR}=\ln\left(\bar{X_{t}}/\bar{X_{c}}\right)$$

where $\bar{X_{t}}$ and $\bar{X_{c}}$ are the mean values of a selected variable (e.g., aboveground production, root biomass, and SOC) under K addition and control treatments, respectively.

The variance for meta‐analysis was calculated as follows:

(2) $$\text{Variance}=\frac{{S_{c}}^{2}}{{\bar{X_{c}}}^{2}\;n_{c}}+\frac{{S_{t}}^{2}}{{\bar{X_{t}}}^{2}\;n_{t}}$$

where $s_{c}$ and $s_{t}$ are the standard deviations for control and treatment, respectively; $\bar{X_{c}}$ and $\bar{X_{t}}$ are the average values for control and treatment, respectively; and $n_{c}$ and $n_{t}$ are the sample sizes for control and treatment, respectively.

The percentage change (%) of K effects was evaluated as:

(3) $$\text{Percentage change}=\left(e^{\mathrm{WRR}}–1\right)\times 100$$

where WRR is the weighted RR.

The package named “metafor” was used to perform a random‐effects model to conduct the meta‐analyses. To determine the potential publication bias, we created funnel plots using “funnel” function and performed the Egger test using “regtest” function. No significant publication bias was found (Figure S4).

By using the “rma” function under the package “metafor” in R, we determined the relationships between RR and climate variables, N, P, and K application rate, experimental duration, and all initial soil variables. For studies reporting both plant production (aboveground production and root biomass) and SOC under both control and K treatments, the same statistical method was applied to determine the relationship between the RR of SOC and plant production. In this way, we could assess whether K can increase SOC by enhancing plant production.

In order to directly test whether N and/or P limitations could be alleviated by N and/or P addition and whether this would significantly change K effects, we filtered datasets from the fully factorial nutrient addition experiments (e.g., N and K, P and K, and N, P, and K). Regarding aboveground production, 102, 101, and 105 studies were collected for N and K, P and K, and N, P, and K fully factorial experiments, respectively; regarding root biomass, 26, 27, and 28 studies were collected for N and K, P and K, and N, P, and K fully factorial experiments, respectively; and regarding SOC, 28, 32, and 28 studies were collected for N and K, P and K, and N, P, and K fully factorial experiments, respectively. The means and standard deviations under each treatment combination were also collected to conduct the meta‐analysis by using the aforementioned method.

We identified the potential N, P, and NP‐limited ecosystems by using the RRs of N (N vs. control), P (P vs. control), and NP (NP vs. control). More specifically, we reviewed all papers that were included in this meta‐analysis to filter the studies reporting the significance of N and/or P effects on aboveground productivity. Since very few studies reported the significance of N and/or P effects on root biomass (0) and soil organic carbon (2), we were not able to conduct the analyses for these two variables. Regarding aboveground productivity, 23 out of 102, 16 out of 101, and 18 out of 105 studies reported the significance of the effects of N, P, and NP, respectively. We then utilized the studies that showed insignificant effects of N, P, or NP on aboveground productivity to calculate mean values of the RRs of N, P, and NP that would be used as the thresholds to determine whether the ecosystems were limited by N, P, and NP. Specifically, if the RRs of N, P, and NP were lower than 0.204, 0.0422, and 0.212, the ecosystems were not limited by N, P, and NP, respectively. It should be noted that the method for defining nutrient‐limited ecosystems can be arbitrary, especially when only approximately 20% of studies reported the significance of the effects of N, P, and NP.

The boosted regression tree (BRT) analysis was performed in R with “gbm” package to determine the relative influences of predictor variables on K effects on aboveground production, root biomass, and SOC. Parameter values used for the BRT analysis, including shrinkage, interaction.depth, and ntree, were set at 0.01, 2, and 12,000, respectively. To ensure the BRT analysis's accuracy, only variables reported by more than 80% of studies were included. Specifically, the response variable was RR, and the predictor variables consisted of climate variables (MAT, MAP, and AI), K fertilizer type (KCl and K2SO4) and application rate, K addition scenario (e.g., K vs. control, NK vs. N, PK vs. P, and NPK vs. NP), experimental duration (1–3 years and > 3 years), and ecosystem type (forests, grasslands, wetlands, and tundra). Overall, the BRT analysis explained 34% of the variation in the RR of aboveground productivity to K, 41% in the RR of root biomass to K, and 32% in the RR of SOC to K. Although some of the initial soil variables (e.g., SOC, soil total N, pH, clay content, and bulk density) were extracted from the database “SoilGrids” according to the locations of the studies, almost all of them were not able to explain K effects on plant production and SOC (Table 1). In other words, N and P addition can significantly improve soil N and P content, which should be a better indicator of soil nutrient availability than the estimated values from “SoilGrids.” Therefore, we did not include initial soil parameters as predictor variables in the BRT analysis. All statistical analyses and graphs were performed using R Studio (version 2024.04.0+735).

## 3 Results

### 3.1 K Effects on Aboveground Production

**FIGURE 1:**
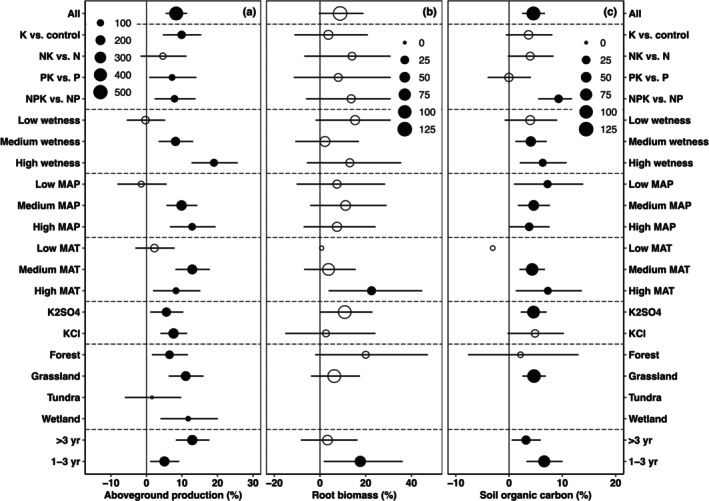
Effects of potassium on aboveground production (a), root biomass (b), and soil organic carbon content (c). The percentage changes (mean ± 95% confidence intervals [CI]) were shown. The size of each point is proportional to the sample size. Open and solid points represent insignificant and significant effects of potassium, respectively. The Low MAT in panels (b) and (c) had large 95% CIs, which were not shown for better visualization. Wetness includes low (aridity index [AI] < 0.5), medium (0.5–1.0), and high (AI > 1.0). Mean annual precipitation (MAP) includes low (< 500 mm), medium (500–1000 mm), and high (> 1000 mm). Mean annual temperature (MAT) includes low (< 5°C), medium (5°C–10°C), and high (> 10°C). K addition scenarios include K versus control (comparing K addition treatment with no nutrient addition treatment), NK versus N (comparing treatment under both N and K addition with N addition treatment), PK versus P (comparing treatment under both P and K addition with P addition treatment), and NPK versus NP (comparing treatment under N, P, and K addition with treatment under both N and P addition). K type includes K2SO4 and KCl, ecosystem type includes forest, grassland, wetlands, and tundra, and experimental duration includes 1–3 years and > 3 years.

**FIGURE 2:**
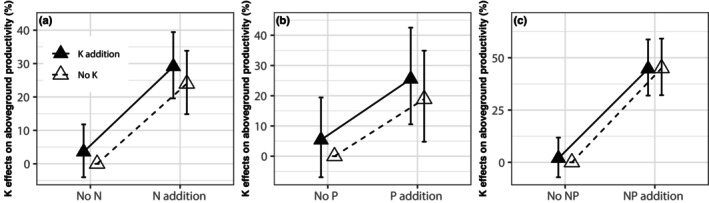
Effects of nutrient addition on aboveground production from fully factorial experiments including nitrogen (N) and potassium (K) (a), phosphorus (P) and K (b), and N, P, and K (c). The percentage changes (mean ± 95% confidence intervals [CI]) were shown.

On average, across all observations, we found that added K (of 140 kg ha^−1^ year^−1^ on average) increased aboveground production by 8% (*p* < 0.001, Figure 1a). K increased aboveground production the most (10%, *p* < 0.001, K vs. control) in the absence of the addition of N, P, or both (Figure 1a), and increased it slightly less when N (5%, *p* = 0.15, NK vs. N), P (7%, *p* < 0.05, PK vs. P), or both (8%, *p* < 0.01, NPK vs. NP) were added to both control and K addition treatments. Insignificant differences were found between K addition scenarios (e.g., K vs. control, NK vs. N, PK vs. P, and NPK vs. NP). By analyzing the dataset from the fully factorial nutrient (N, P, and K) addition experiments, we found that N and/or P could significantly increase aboveground production (Figure 2), demonstrating N and/or P limitations in the experiments. However, the positive effect of K on aboveground production was not further amplified by alleviating N and/or P limitations (Figure 2), which is not consistent with Hypothesis 1. Our results also showed that K effects on aboveground productivity were not significant in non‐N, non‐P, or non‐NP limited ecosystems (Figure S5), which further supports our finding: alleviating N and P limitation does not amplify K‐induced increase in aboveground productivity. By contrast, K effects on aboveground productivity were positive in N or P‐limited ecosystems, indicating that the regions that are limited by N or P are also limited by K.

As we expected in Hypothesis 2, K addition did not show a significant effect on aboveground production in low wetness, MAP, or MAT (Figure 1a). By contrast, the greatest increase in production was found under high wetness or MAP, indicating that plant growth is more K‐limited in wet regions. The effect of K on aboveground production was positive in forests (+6%; 95% CI: 2%–12%), grasslands (+11%; 95% CI: 6%–16%), and wetlands (+12%; 95% CI: 4%–20%), but neutral in tundra (+2%; 95% CI: −6% to 10%). K addition increased aboveground production by 5% and 13% in the short‐term (1–3 years; *p* < 0.05) and long‐term (> 3 years; *p* < 0.001), respectively (Figure 1a).

**FIGURE 3:**
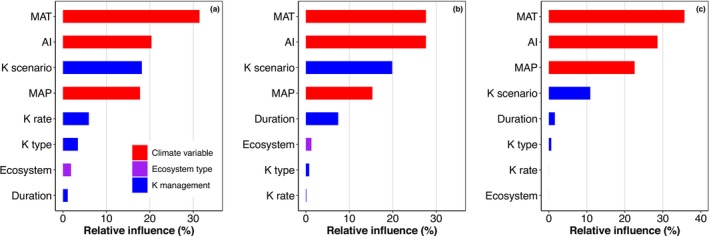
The relative contributions (%) of predictor variables for the boosted regression tree model of response ratio of aboveground production (a), root biomass (b), and soil organic carbon content (c). AI: Aridity Index; Duration: experimental duration; Ecosystem: ecosystem type; K rate: K application rate; K scenario: K fertilizer management including K versus control, N versus NK, P versus PK, and NP versus NPK; K type: K fertilizer type; MAP: mean annual precipitation; MAT: mean annual temperature.

According to the BRT analysis, K effects on aboveground production were mostly explained by MAT (31%), AI (20%), K addition scenario (e.g., whether K was added together with N, P, or NP, 18%), and MAP (18%, Figure 3a). Moreover, K application rate, K fertilizer type, ecosystem type, and experimental duration explained 6%, 4%, 2%, and 1% of the variation in K effects on aboveground production, respectively.

### 3.2 K Effects on Root Biomass

As predicted in Hypothesis 3, K modestly but not significantly increased root biomass by 9% (*p* < 0.1, Figure 1b). K effects on root biomass were mediated by MAT and experimental duration (Figure 1b). For example, a significant increase in root biomass caused by K was only found under high MAT. K was also found to stimulate root biomass only in the short term (e.g., 1–3 years), which was inconsistent with what we found for aboveground production. Alleviating N and/or P limitation did not further change K effects on root biomass (Figure S6a–c). The BRT analysis showed that K effects on root biomass were mostly explained by climate variables (e.g., MAT: 28%, AI: 28%, and MAP: 15%; Figure 3b).

### 3.3 K Effects on Soil Organic Carbon

**FIGURE 4:**
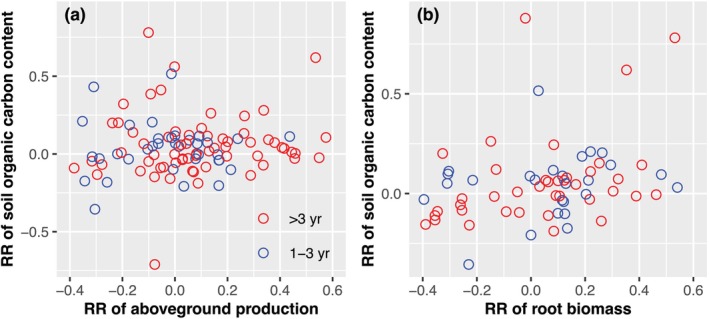
Relationships between response ratios of soil organic carbon, aboveground production (a, *N* = 116), and root biomass (b, *N* = 103) under different experimental durations (1–3 years and > 3 years). Insignificant (*p* > 0.1) relationships were found, regardless of experimental duration.

K addition led to a modest but significant 5% increase in SOC, with the effects varying depending on climate variables, K type, application scenarios, and ecosystem types (Figure 1c). Specifically, a significant 9% increase in SOC caused by K addition was observed only when N and P were added under both control and K treatments, which was also supported by the results from the fully factorial nutrient addition experiments (Figure S6f). Moreover, K effects on SOC were insignificant under low wetness and MAT, but became positive under medium and high wetness and MAT. SOC showed a significant response to K only when K fertilizer type was K_2_SO_4_. Partly because of the small studies in forests, we only found a K‐induced increase in SOC in grasslands. Overall, 86% of the variations in K effects on SOC were explained by climate variables including MAT, AI, and MAP (Figure 3c). No significant relationships were found between RRs of SOC and those of aboveground productivity (Figure 4a) or root biomass to K (Figure 4b), which is inconsistent with Hypothesis 4.

## 4 Discussion

### 4.1 Alleviating N and P Limitation Does Not Amplify K‐Induced Increase in Aboveground Productivity

As hypothesized, we found a positive effect of K on aboveground production. It should be noted that, across the entire data set, the increase in aboveground production caused by added K (8%) was smaller than that caused by added N (34%) (Lebauer and Treseder 2008) and added P (35%) (Hou et al. 2020). However, as almost no published studies reported the proportional increase in N, P, or K availability due to experimental additions, nor do we have a framework for interpreting the biogeochemical or plant physiological response to a given addition magnitude, comparing different responses among elements is difficult. Regardless, even a moderate increase in aboveground production under K addition (8% overall) suggests that K limitation is important globally to C cycling, and K addition could hypothetically be a tool for increasing production and fighting climate warming.

It was surprising, however, and inconsistent with our hypothesis, that K effects on aboveground productivity should be greater when N and P are not limiting. When we group sites by N and/or P limitation (Figure S5), we found positive K effects on aboveground productivity only in systems limited by N or P. This suggests that K could somehow substitute for N or P when they were limiting. In other words, a positive effect of K on aboveground productivity does not require N or P limitation, and K limitation does not grow larger when N or P limitation is alleviated. Furthermore, K effects on aboveground productivity may in fact be greater or more frequent when N and P are not limiting. If this result is a general pattern, ESMs would therefore need to represent K limitation separately from N and P limitation to predict ecosystem response to nutrient limitations. Overall, the role of K in improving aboveground production is likely mediated by its impacts on the water economy, but more N and P supply can also improve water use efficiency (Brueck 2008; Khan et al. 2023), so the key role of K in enhancing production can be diminished under a high amount of N and P. In addition, although N can generally increase plant production, indirect negative effects (e.g., soil acidification and nutrient loss) on plant growth can appear and worsen over time, interfering with K effects. For example, a global meta‐analysis found that N‐addition decreased soil pH by 0.25 and 0.34 after 1–5 and 5–10 years in terrestrial ecosystems, respectively (Tian and Niu 2015). Soil acidification caused by N application also led to decreases in soil exchangeable K^+^, Ca^2+^, and Mg^2+^. In addition, by analyzing 44 experimental studies in terrestrial ecosystems, a previous study (Tian et al. 2016) found that N saturation of terrestrial ecosystem net primary productivity appeared when N application rate was higher than 50–60 kg ha^−1^ year^−1^. For our study, the average N application rate was 163 N kg ha^−1^ year^−1^ across 220 studies; moreover, 127 out of 232 N and NP studies have been conducted for more than 3 years. As a result, the long‐term addition of large amounts of N in these studies might ultimately offset positive K effects on aboveground production by causing soil acidification and N saturation and decreasing nutrients (e.g., K, Ca, and Mg) uptake. Similarly, in addition to weakening the roles of K in water use efficiency enhancement, high P levels can inhibit K, calcium, iron, copper, and zinc uptake (Ayamba et al. 2023). P was applied at a relatively high rate (average value: 120 kg P ha^−1^ year^−1^) in the experiments that were included in the present study. Consequently, K effects on aboveground production were a little bit smaller when P was applied under both control and K treatments than when K was applied alone.

As we expected in Hypothesis 2, K limitation of aboveground production became more pronounced with increasing MAP and decreasing aridity. In other words, aboveground production is more K‐limited in wet regions. This might be because, under arid conditions, K is more focused on water saving and stress avoidance (e.g., resistance and resilience); under favorable water conditions, however, K can improve conditions (C assimilation and storage) except for water economy, favoring more suitable plant production. Moreover, K is highly soluble in soils and is the most rapidly released nutrient from litter during decomposition (Sardans and Peñuelas 2021), which means that K can be leached more easily under wet than dry conditions, leading to more serious K limitation in wet regions. In addition, high vegetation production and thus greater plant K demand in wet than dry regions might also lead to a larger increase in aboveground production stimulated by K addition.

### 4.2 The Modest Effect of K on Belowground C Stock

The modest increase in root biomass (9%, *p* < 0.1) caused by K addition suggests that K generally tends to stimulate above‐ more than below‐ground production in terrestrial ecosystems. We found that K effects on root biomass were not significant under low and medium MAT but became positive under high MAT, reflecting that not only above‐ but also below‐ground production is more K‐limited in warm regions. We found a contrasting effect of experimental duration on the response of aboveground productivity and root biomass to K. Specifically, a positive effect of K was only found in the short term for root biomass. This finding indicates that, when compared to aboveground, belowground production is more likely to become K‐saturated in the mid term. Overall, the insignificant K effects on root biomass suggest the multiple mechanisms that can stimulate or inhibit root growth might offset each other. Almost all studies did not measure and report water use efficiency and plant nutrient requirements under K addition, making it difficult to explicitly explain why K did not significantly affect belowground production. In order to unravel the mechanisms of how K affects root growth, more studies that measure all potential processes are required in the future.

The minor but significant increase in SOC (5%) under K indicates that K addition plays a great role in terrestrial C sequestration and thus climate mitigation, given that soils contain more C than the atmosphere and vegetation combined. Similar to aboveground productivity, SOC showed a positive response to K only in regions with relatively high wetness and MAT, indicating that not only aboveground productivity but also SOC is K‐limited more in tropical ecosystems. K effects on SOC were significantly positive only when both N and P were added to control and K treatments. This might be due to the higher litterfall quality and soil C use efficiency (CUE) induced by N and P addition. For example, some field studies found that, when compared to N or P addition, combined N and P addition improved both the quantity (O'Connell and Grove 1993) and quality (Tie et al. 2023) of litterfall in forests. Therefore, a K‐induced increase in litterfall might have a greater quality (e.g., lower C/N and lignin/N) under both N and P addition, and thus accelerate the release of litter C, N, and micronutrient (Tie et al. 2023), ultimately stimulating CUE (Cotrufo et al. 2013). The chloride ion in KCl has direct toxicity on soil microbes and can decrease soil N availability by inhibiting nitrification (Khan et al. 2014); therefore, KCl‐induced soil microbial stress might lead to a lower CUE, which in turn decreases SOC accumulation. As a result, K effects on SOC were insignificant when K type was KCl.

It should be noted that the insignificant differences in K effects on SOC between short‐ (1–3 years) and long‐term (> 3 years) found in this study do not necessarily mean that the role of K in SOC sequestration would not change over time. It takes a relatively long time for the litterfall to be decomposed (Cai et al. 2020) and stabilized in soil; however, the limited number of long‐term studies reporting SOC under K (i.e., the median experimental duration of studies collected in this meta‐analysis was 5 years) makes it difficult for us to determine long‐term effects of K on SOC. Another global meta‐analysis that we are doing for croplands showed that K effects on SOC were insignificant in the short term (1–20 years; 101 studies) but became positive in the long term (> 20 years; 152 studies, unpublished data). This suggests that the K‐induced increase in SOC may be stronger after a long time of K addition.

## 5 Conclusions, Limitations, and Implications

The importance of production in terrestrial ecosystems being often N limited has long been a focus (Vitousek and Howarth 1991), and similar emphasis on P limitation has also grown in recent decades (Du et al. 2020; Hou et al. 2020). When compared to N and P, however, the effects of K on terrestrial production and C stocks (especially the belowground) have been generally overlooked. To the best of our knowledge, this may be the first broad assessment of the responses of both above‐ and below‐ground C stocks to K addition in terrestrial ecosystems. The K limitations of aboveground production and SOC that were found in the present study indicate that K addition could be an important nutrient management tool, which could additionally be implemented to increase C sequestration in terrestrial ecosystems. It should be noted that, however, adding K does not necessarily lead to a greater increase in aboveground production in ecosystems that are not N and/or P‐limited. By overlooking these, we may overestimate the roles of K addition in sequestrating C in terrestrial ecosystems.

Although our study provides a large and unparalleled dataset about terrestrial production and SOC under K addition at the global scale, there is a large uncertainty in our estimate of K effects on terrestrial ecosystems. First, for example, the various measures of aboveground plant production might result in uncertainties. Therefore, measuring and reporting the same type of aboveground production across experiments would be essential for us to better estimate the response of aboveground plant production to K addition. It should be noted that, however, the main conclusions of this study should not be significantly affected by this issue. For example, we divided the measures of aboveground plant production into two types (productivity [*N* = 214] and biomass [*N* = 314]), and the overall effect of K on aboveground production did not show a significant difference between productivity and biomass (Figure S3). Second, although our dataset includes some studies in tropical regions, ecosystems in the tropics are still largely underrepresented. Since our results suggest that K limitation is larger or more frequent in wet regions, more studies from the tropical zones would likely increase our estimated global averaged K‐induced increase in terrestrial C. Third, it takes a relatively long time for litterfall to be decomposed and transferred to soil C. The time difference between these two processes might lead to insignificant relationships between K effects on SOC and aboveground productivity and root biomass.

It is understandable that ESMs have considered the roles of N and P but not K in global C cycling because of the limited data about K effects. However, the K limitations of terrestrial aboveground production and SOC that we found call for more studies about K effects in order to generate a better understanding of the macronutrient limitation of terrestrial ecosystems. It should be noted that few studies of K addition reported initial soil nutrient availability (e.g., 148, 33, 12, 48 out of 528 studies for soil total N, total K, available N, and available K, respectively). In addition, relevant variables (e.g., photosynthesis, litterfall quantity and quality, resource use efficiency, and soil biochemical parameters) that are involved in the processes relating to K effects on plant production and SOC were rarely provided. For example, among 528 studies reporting aboveground production, only 0, 17, 4, 0, 17, and 12 studies provided soil microbial biomass C, total N and K, available N and K, pH, and cation exchange capacity under both control and K addition treatments, respectively. Measuring more of the relevant parameters would be important for enabling further unraveling of the mechanisms of how K affects C cycling in terrestrial ecosystems.

## Author Contributions

**Guopeng Liang:** conceptualization, data curation, formal analysis, methodology, resources, visualization, writing – original draft, writing – review and editing. **Pengyan Sun:** data curation. **Bonnie G. Waring:** writing – review and editing. **Zheng Fu:** writing – review and editing. **Peter B. Reich:** writing – review and editing.

## Conflicts of Interest

The authors declare no conflicts of interest.

## Supporting information


Data S1.

## Data Availability

The datasets were archived in figshare (https://doi.org/10.6084/m9.figshare.28644407.v1).
