# Supplementary material for: Alleviating Nitrogen and Phosphorus Limitation Does Not Amplify Potassium‐Induced Increase in Terrestrial Biomass
Source: Glob Chang Biol. 2025 Apr 23;31(4):e70193. doi: 10.1111/gcb.70193 (PMC12018727; doi:10.1111/gcb.70193)
Supplement: Supplementary file 1 — Data S1. [file GCB-31-e70193-s001.docx]

**Supplementary Materials for**

**Alleviating nitrogen and phosphorus limitation does not amplify potassium-induced increase in terrestrial biomass**

Guopeng Liang *et al.*

*Corresponding authors: Guopeng Liang ([Guopeng.Liang@yale.edu](mailto:Guopeng.Liang@yale.edu)); Peter B. Reich ([preich@umich.edu](mailto:preich@umich.edu))

******Supplementary Figure 1** PRISMA flow diagram showing the procedure used for the selection of studies for synthesis. (a) plant production and (b) soil organic carbon.

**Supplementary Figure 2** Location of the field potassium addition experiments selected in this study. (a) aboveground production; (b) root biomass; and (c) soil organic carbon.

**Supplementary Figure 3** Effects of potassium on aboveground production under different types (biomass and productivity). The percentage changes (mean ± 95% confidence intervals [CI]) were shown. The number next to the dots is the sample size of each variable.

******Supplementary Figure 4** Funnel plots for potassium effects. Results of publication bias tests using Egger's regression are given at the top of each panel (z and p values). p > 0.05 indicates the absence of publication bias. (a) aboveground production; (b) root biomass; and (c) soil organic carbon.

**Supplementary Figure 5 Potassium effects on aboveground production in ecosystems which are limited or not limited by nitrogen and/or phosphorus.** The percentage changes (mean ± 95% confidence intervals [CI]) were shown.

**Supplementary Figure 6 Effects of nutrient addition on root biomass (a-c) and soil organic carbon content (d-f) from fully factorial experiments including nitrogen (N) and potassium (K), phosphorus (P) and K, and N, P (NP), and K.** The percentage changes (mean ± 95% confidence intervals [CI]) were shown.

| Variable | Forest | Grassland | Wetland | Tundra |
| --- | --- | --- | --- | --- |
| **Aboveground** |  |  |  |  |
| Aboveground biomass | 12 | 129 | 68 | NA |
| Aboveground net primary production | 7 | 100 | NA | NA |
| Basal/volume/wood growth | 105 | NA | 8 | NA |
| Relative growth rate | 13 | NA | NA | NA |
| Tree height | 26 | NA | NA | NA |
| Leaf/shoot biomass | 9 | NA | NA | 51 |
| **Belowground** |  |  |  |  |
| Root biomass | 18 | 107 | NA | NA |
| Soil organic carbon | 9 | 117 | NA | NA |

**Supplementary Table 1** Summary of measurements of plant production and soil organic carbon collected in this meta-analysis study and the corresponding number of studies

NA: not applicable.
